# Avoiding entry into intracellular protein degradation pathways by signal mutations increases protein secretion in *Pichia pastoris*

**DOI:** 10.1111/1751-7915.14061

**Published:** 2022-06-03

**Authors:** Yoichiro Ito, Misa Ishigami, Noriko Hashiba, Yasuyuki Nakamura, Goro Terai, Tomohisa Hasunuma, Jun Ishii, Akihiko Kondo

**Affiliations:** ^1^ 13593 Engineering Biology Research Center Kobe University 1‐1 Rokkodai, Nada Kobe 657‐8501 Japan; ^2^ 13593 Graduate School of Science, Technology and Innovation Kobe University 1‐1 Rokkodai, Nada Kobe 657‐8501 Japan; ^3^ Technology Research Association of Highly Efficient Gene Design (TRAHED) Kobe Japan; ^4^ 13143 Department of Computational Biology and Medical Sciences Graduate School of Frontier Sciences The University of Tokyo Chiba Japan; ^5^ 13593 Department of Chemical Science and Engineering Graduate School of Engineering Kobe University Kobe Japan; ^6^ 13593 Center for Sustainable Resource Science RIKEN Yokohama Japan

## Abstract

In our previous study, we serendipitously discovered that protein secretion in the methylotrophic yeast *Pichia pastoris* is enhanced by a mutation (V50A) in the mating factor alpha (MFα) prepro‐leader signal derived from *Saccharomyces cerevisiae*. In the present study, we investigated 20 single‐amino‐acid substitutions, including V50A, located within the MFα signal peptide, indicating that V50A and several single mutations alone provided significant increase in production of the secreted proteins. In addition to hydrophobicity index analysis, both an unfolded protein response (UPR) biosensor analysis and a microscopic observation showed a clear difference on the levels of UPR induction and mis‐sorting of secretory protein into vacuoles among the wild‐type and mutated MFα signal peptides. This work demonstrates the importance of avoiding entry of secretory proteins into the intracellular protein degradation pathways, an observation that is expected to contribute to the engineering of strains with increased production of recombinant secreted proteins.

## Graphical abstract

Several single‐amino‐acid substitutions, including a serendipitously discovered mutation V50A, located within the mating factor alpha (MFα) prepro‐leader signal peptide provided significant increase in production of the secreted proteins in *Pichia pastoris*. Our unfolded protein response (UPR) biosensor analysis and microscopic observation showed a clear difference on the levels of UPR induction and mis‐sorting of secretory protein into vacuoles among the wild‐type and mutated MFα signal peptides, indicating that the importance of avoiding entry of secretory proteins into the intracellular protein degradation pathways lead to the engineering of strains with increased production of recombinant secreted proteins.

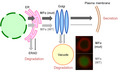

## Introduction

*Komagataella phaffii*, commonly known as *Pichia pastoris*, is an important platform for recombinant protein production, for both academic and industrial purposes. This yeast has a number of attractive features, including high protein secretion capacity, powerful high cell‐density cultivation and good post‐translational modification, in addition to the availability of extraordinarily strong and tightly methanol‐regulated promoters (Damasceno *et al*., 2012; Gasser *et al*., 2013; Ahmad *et al*., 2014; Puxbaum *et al*., 2015; Fischer and Glieder, 2019). These properties have permitted the use of *P. pastoris* in the successful production of multiple recombinant proteins, although some heterologous secreted proteins have been difficult to overproduce, typically depending on the nature of the produced protein (Puxbaum *et al*., 2015). To overcome this difficulty, many studies have been performed in the effort to improve the production of such proteins. Associated strategies have included genetic modification of *P. pastoris* strains, as well as the optimization of sequences (promoters, protein secretion signals and terminators) that regulate expression of target genes (Delic *et al*., 2014; Vogl *et al*., 2016; Barrero *et al*., 2018; Ito *et al*., 2020).

Given its critical role in regulating recombinant protein production, the secretion signal has been a focus of attempts at optimization (Damasceno *et al*., 2012; Gasser *et al*., 2013; Ahmad *et al*., 2014; Heiss *et al*., 2015; Puxbaum *et al*., 2015). Among the secretion signals, the *Saccharomyces cerevisiae* α‐mating factor prepro‐leader (MFα signal peptide) is widely used for recombinant protein production in *P. pastoris* (Damasceno *et al*., 2012; Gasser *et al*., 2013; Ahmad *et al*., 2014). The MFα signal peptide consists of a pre‐region (19 amino acids) followed by pro‐region (66 amino acids) including a C‐terminal dibasic Kex2 endopeptidase cleavage site (KR). The pre‐region has a typical signal structure and directs translocation from the cytoplasm to the lumen of the endoplasmic reticulum (ER). The pro‐region is thought to facilitate the proper transit of target polypeptides from the ER to the Golgi apparatus via a coat protein complex II (COPII)‐dependent pathway (Otte and Barlowe, 2004; Fitzgerald and Glick, 2014). Recently, it also was reported that the pro‐region of the wild‐type MFα signal peptide is prone to aggregation in the ER, which may impede the secretion of recombinant protein (Barrero *et al*., 2018). To improve the production of recombinant proteins, researchers have targeted the MFα signal peptide in *P. pastoris* or *S. cerevisiae* using several strategies, including codon optimization (Ahn *et al*., 2016), directed evolution (Rakestraw *et al*., 2009; Aza *et al*., 2021), large‐deletion mutagenesis (Lin‐Cereghino *et al*., 2013; Chahal *et al*., 2017) and the construction of chimeric signal sequences combining those of Ost1 and MFα pro‐region (Fitzgerald and Glick, 2014; Barrero *et al*., 2018; Besada‐Lombana and Da Silva, 2019).

Recently, we discovered, in the course of a genome‐wide screening for *P. pastoris* mutants, that a V50A mutation in the MFα signal peptide leads to increased secretion of a recombinant reporter protein (Ito *et al*., Submitted). Compared to the wild‐type MFα signal peptide, a peptide harbouring the V50A mutation resulted in an approximately six‐fold improvement in secretion of an anti‐lysozyme single‐chain variable fragment (scFv) antibody, an effect that was stronger than those (1.2‐ to 1.6‐fold) typically observed with the gene‐disruption events that were being screened. This result suggested that this single‐amino‐acid substitution in the MFα signal peptide may overcome a rate‐limiting step in the yeast protein secretion pathway.

In the present study, we further characterized (in *P. pastoris*) V50A, along with several other single‐amino‐acid substitutions in the MFα signal peptide that have been reported to improve the secretion of various recombinant proteins in *S. cerevisiae*. Although there were differences, depending on the reporter proteins and expression modes, combinations of a small number of mutations yielded similar or slightly higher levels of protein secretion under various test conditions. In contrast, decreases in the protein secretion titres often were observed when a large number (> 3) of MFα signal peptide mutations were combined. Further work employing an unfolded protein response (UPR) biosensor analysis and microscopic observation using constructs encoding secreted green fluorescent protein (GFP) suggested that the improvements in secretion may be attributable to the prevention of UPR activation and mis‐sorting to vacuoles, both which would direct recombinant proteins to intracellular protein degradation pathways.

## Results and discussion

### Identification of novel effective single‐amino‐acid substitutions in the MFα signal peptide

In our previous work, genome‐wide screening for enhanced secretion of a small antibody was performed using a random gene‐disruption library of the *P. pastoris* CBS7435 strain (Ito *et al*., Submitted). The anti‐lysozyme scFv antibody is a model protein that has been difficult to produce in secreted form. Therefore, we designed plasmids encoding scFv harbouring an N‐terminal MFα prepro secretion leader peptide (MFα signal peptide) and a C‐terminal His_6_‐tag. The *scFv* gene was expressed under the control of the methanol‐inducible *AOX1* promoter. In this screen, a yeast strain with elevated secretion of the scFv antibody was obtained. Characterization of this mutant strain revealed that the increased secretion resulted not from a genomic gene‐disruption event, but serendipitously from a novel mutation in the reporter plasmid (Ito *et al*., Submitted). Specifically, we found that the scFv reporter protein now was encoded with a single amino acid substitution (V50A) in the MFα signal peptide, and that this change was sufficient to provide enhanced production of the secreted recombinant protein.

**Fig. 1:**
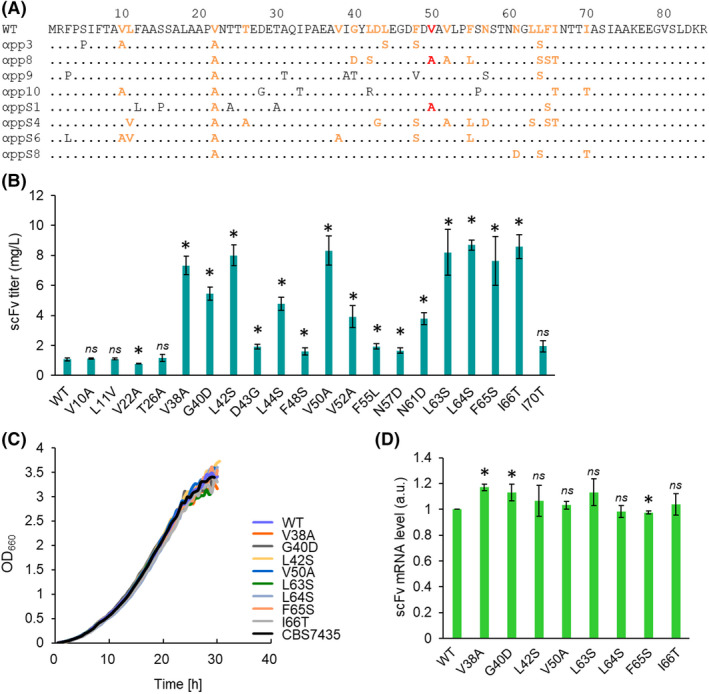
Properties of *Pichia pastoris* strains harbouring *AOX1* promoter‐driven constructs encoding scFv with single‐amino‐acid substitutions in the MFα signal peptide. A. Positions of single‐amino‐acid substitutions in the MFα signal sequence used in this study. Amino acid mutations of evolved MFα signal peptide mutants obtained by directed evolution in *S. cerevisiae* (Rakestraw *et al*., 2009) are shown (WT: wild‐type MFα signal). Coloured residues indicate the positions of V50A (red) and the other amino acid mutations (orange) characterized in the present study. The yeast strains encoding scFv with an MFα signal peptide containing each of the single‐amino‐acid substitutions at the 19 indicated residues (orange) were newly constructed and evaluated. B. scFv titres of the single‐amino‐acid substitution (and WT) strains. The cells expressing scFv were cultured in BMMY (methanol) medium. The scFv titres were determined by an ELISA assay. Strains containing each of 20 substitutions, as well the wild‐type MFα sequence, are indicated. Bars and circles indicate scFv titres and final optical density at 660 nm (OD_660_) values respectively. C. Growth rate analysis in BMMY. Growth curves of the eight strains (including V50A) with the highest scFv titres (coloured symbols), as well as the host strain (CBS7435; black), are indicated. D. *scFv* mRNA levels of the *P. pastoris* strains. The cells expressing scFv under the control of the *AOX1* promoter were cultured in BMMY for 6 h. The strains used were the same as in (C). Values are plotted as mean ± standard deviation of three biological replicates. Asterisks indicate significance (*p* < 0.05 by *t*‐test) between the wild‐type MFα signal strain and the mutant strains in (B and D).

The V50A mutation had been reported previously as a component (in combination with other mutations) of a set of MFα signal mutants discovered as part of a directed evolution experiment in *S. cerevisiae* (Rakestraw *et al*., 2009) (Fig. 1A). In that work, two mutants containing the V50A mutation were identified among eight evolved MFα signal mutants (Fig. 1A). This observation motivated us to explore the possibility that other amino acid substitutions identified among the previously reported directed‐evolution MFα mutants also might enhance protein secretion in our system. We therefore chose to screen the other 19 amino acid mutations described by Rakestraw *et al*. for their effects on protein secretion in our assay (Fig. 1A). We constructed *P. pastoris* strains in which scFv was encoded with each of 20 single‐amino‐acid substitutions (including V50A) in the MFα signal peptide and evaluated the scFv secretion ability of each strain (Fig. 1B). Colony PCR and qPCR were performed to verify that each of these strains harboured a single‐copy insertion at the targeted locus in the *P*. *pastoris* genome, ensuring a meaningful comparison.

We found that seven of the single‐amino‐acid substitutions in the MFα signal peptide (V38A, G40D, L42S, L63S, L64S, F65S and I66T respectively), as well as the V50A mutation, exhibited notable (more than five‐fold) increases in the level of production of secreted scFv compared to that seen with the wild‐type MFα signal peptide (Fig. 1B). We further analysed strains harbouring these single‐amino‐acid substitutions in the MFα signal peptide by assaying their growth rates and the levels of the mRNAs encoding the scFv protein when grown under inducing conditions (in methanol‐containing BMMY medium). All eight single‐amino‐acid substitution strains appeared growth rates comparable to those of a strain harbouring the wild‐type construct (Fig. 1C). The *scFv* mRNA levels in the eight substitution strains appeared similar to those of the strain harbouring the wild‐type construct (Fig. 1D). Given these results, the effects of single‐amino‐acid substitutions on scFv secretion are inferred to be mediated by changes in protein trafficking, rather than changes in cell growth or transcript accumulation.

### Screening and bottom‐up approaches for optimization of secretion signal mutations

To explore the combinations of MFα amino acid substitutions with the best performance, we initially took a combinatorial screening approach. In general, the optimization of fitness for a phenotype obtained by directed evolution typically results in the accumulation of advantageous mutations. To test the potential for further improvement of the scFv secretion ability, we performed a screen using a library containing random combinations of the top‐seven‐ranked amino acid substitutions in the MFα signal peptide (Fig. 1B), that is, containing various combinations of the seven single‐amino‐acid MFα signal peptide mutations that yielded the strongest effects on scFv levels. Again, the strains were designed to express the constructs under the control of the *AOX1* promoter. The high‐throughput screening system constructed in our previous report (Ito *et al*., Submitted) was used to screen the library of combined secretion signal peptide sequences; approximately 900 mutant strains (across ten 96‐well plates) were screened from a combinational library with a diversity of 128 (2^7). However, no variants showed levels of scFv secretion that were notably superior to that seen with the V50A mutation, with the strongest effects being only about 10% higher than that seen with the V50A construct (Fig. S1). This result indicated that combinations of the amino acid substitutions tested here did not provide further enhancement of protein secretion. We inferred that the effects of the single‐amino‐acid mutations all may reflect a shared mechanism.

To confirm that combinations of mutations in the MFα signal peptide indeed do not further enhance protein secretion, we next employed a distinct sequential ‘bottom‐up’ approach. We constructed *P. pastoris* strains by sequential mutation of the MFα‐encoding sequences in constructs under the control of *AOX1* promoter, resulting in the accumulation of combinations of mutations in a given reporter gene. Four constructs harbouring the sequentially generated combinatorial mutations with the eight single amino acid substitutions (V38A, G40D, L42S, V50A, L63S, L64S, F65S and/or I66T) were chosen for testing. These constructs encoded scFv with MFα signal peptides containing mutations as follows: L42S/V50A (a construct designated ‘2xAdv’), L42S/V50A/V38A (‘3xAdv’), L42S/V50A/V38A/L63S (‘4xAdv’) and V38A/G40D/L42S/V50A/L63S/L64S/F65S/I66T (‘8xAdv’). In addition to these four sequentially (cumulatively) mutated constructs, two other constructs encoding scFv with previously reported MFα signal peptide mutations V22A/G40D/L42S/V50A/V52A/F55L/L64S/F65S/I66T (‘αpp8’) and L11V/V22A/T26A/D43G/F48S/V52A/F55L/N57D/L63S/F65S/I66T (‘αppS4’), were generated and screened. These mutated MFα peptides previously had been shown to provide elevated protein secretion in *S. cerevisiae* (Fig. 1A) (Rakestraw *et al*., 2009).

**Fig. 2:**
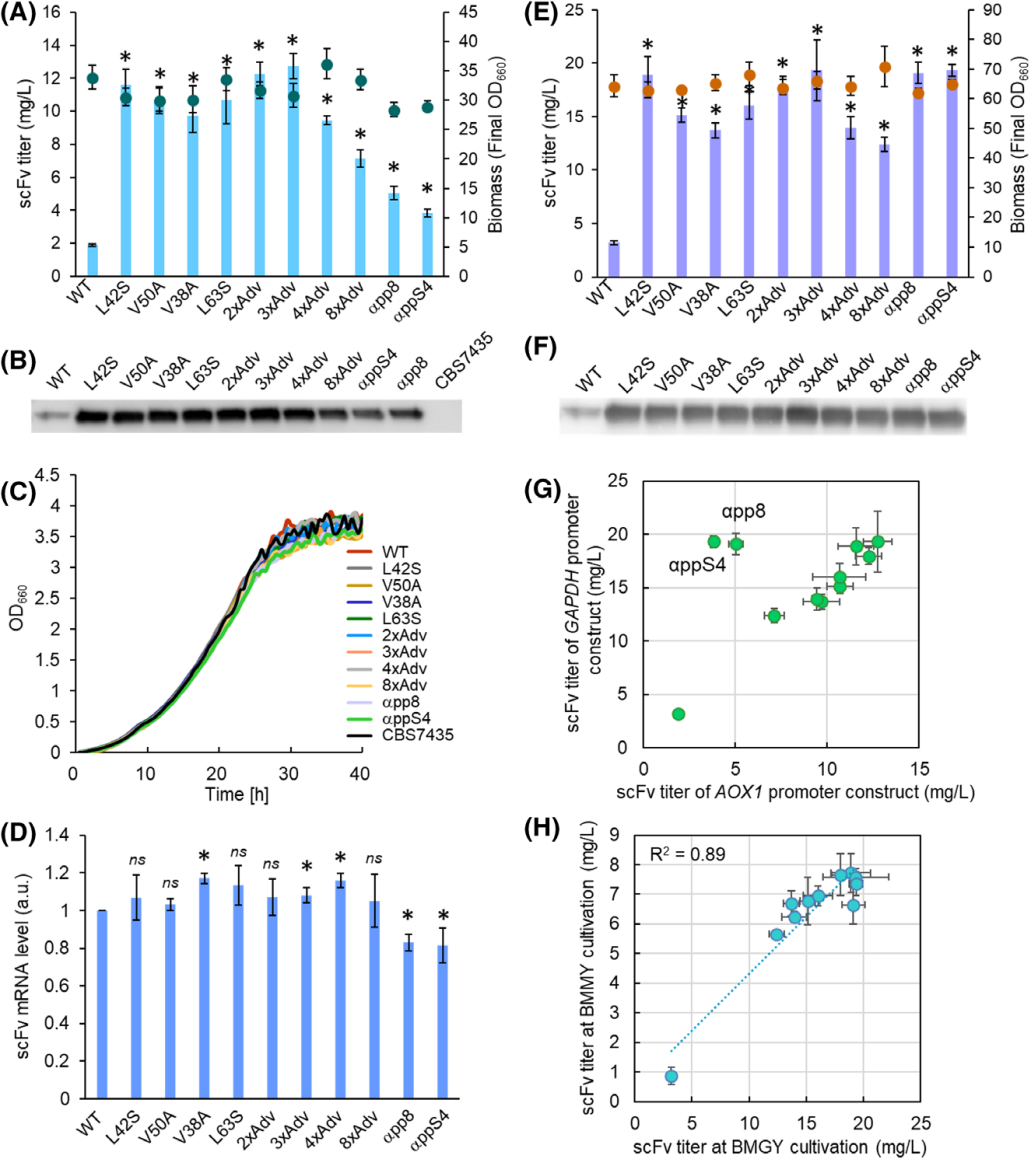
Properties of *Pichia pastoris* strains harbouring constructs encoding scFv with single‐amino‐acid and combined substitutions in the MFα signal peptide. Constructs were under the control of the *AOX1* promoter (A, B, C and D) or the *GAPDH* promoter (E, F). scFv titres (bars) and biomass (final optical density at 660 nm (final OD_660_); circles) (A, E), western blot analyses (B, F), growth curve analysis (C) and *scFv* mRNA levels (D) are shown. The growth rates of various MFα mutation strains (coloured) in BMMY cultivation were compared with that of the host CBS7435 strain (black) (C). (G) Comparison of the scFv titres of the MFα mutant strains containing the *AOX1* promoter constructs (horizontal axis) and the *GAPDH* promoter constructs (vertical axis). (H) Comparison of the scFv titres of the *GAPDH*‐controlled MFα mutant strains cultured in BMGY (glycerol, horizontal axis) and BMMY (methanol, vertical axis). Values are plotted as mean ± standard deviation of three biological replicates. Asterisks indicate significance (*P* < 0.05 by *t*‐test) between the wild‐type MFα signal strain and the mutant strains in (A, D and E).

As shown in Fig. 2A and B, all the mutation‐accumulated strains yielded scFv production titres that were two‐ to six‐fold higher than those obtained with the wild‐type MFα strain. Among the mutation‐accumulated strains, the 2xAdv and 3xAdv strains exhibited scFv levels that were within 10% of those seen with the single‐amino‐acid substitution strains, while the levels with the 8xAdv strain were within 40% lower. Notably, the 4xAdv and 8xAdv strains exhibited a downward trend in the levels of secreted proteins as the number of mutations was increased. The αpp8 and αppS4 strains, which incorporated the literature‐reported mutations in the MFα signal peptide, also showed decreased secretion of reporter protein, compared to the single‐amino‐acid substitution strains.

To clarify these protein secretion trends in the mutation‐accumulated yeast strains, the growth rate in BMMY medium and the *scFv* mRNA levels again were evaluated. Under inducing conditions, the strains encoding the reporter protein with mutation‐accumulated and wild‐type MFα signal peptides showed growth rates that were comparable to that of the CBS7435 host strain (Fig. 2C). Similarly, the *scFv* mRNA levels in the strains encoding the reporter protein with mutation‐accumulated and wild‐type MFα signal peptides were comparable to those seen in the single‐amino‐acid substitution strains (Fig. 2D). On the other hand, the strains encoding the reporter protein with the previously reported MFα signal peptides (αpp8 and αppS4) showed about 20% lower *scFv* mRNA levels than were seen with the wild‐type MFα strain, differences that were statistically significant (*P* > 0.05). This decrease in transcript levels may explain (at least in part) why αpp8 and αppS4 provided lower scFv production in *P. pastoris*.

### Investigation of dependency of the effects of single amino acid substitutions on different promoters and proteins

As mentioned above, we showed that some single‐amino‐acid substitutions in the MFα signal peptide led to higher secretion titres of the scFv antibody under inducing conditions in *P. pastoris*. Here, these MFα signal mutations were further investigated to assess whether the effects of these substitutions were promoter or protein specific. For this purpose, we replaced the inducible *AOX1* promoter driving expression of the *scFv* gene with the constitutive *GAPDH* promoter. Separately, the *scFv* gene under the control of *AOX1* promoter was replaced with each of three other reporter genes: two encoding proteins from other organisms, GFP from the soft coral *Sarcophyton* sp. (mUkG1) (Tsutsui *et al*., 2008; Kaishima *et al*., 2016) and β‐glucosidase (BGL1p) from *Aspergillus aculeatus* (Ito *et al*., 2020); and one encoding an artificial product, blinatumomab, an anti‐CD3/anti‐CD19 bispecific T cell‐engaging tandem scFv antibody (BiTE) (Löffler *et al*., 2000). These protein‐secreting yeast strains carrying the MFα signals with the same amino acid substitution(s) as described in Fig. 2A were newly constructed and evaluated for their protein secretion ability.

In the constitutive protein production constructs (using the *GAPDH* promoter) grown in BMGY (glycerol) medium, the scFv secretion titres of the strains encoding the protein with mutated MFα signal peptides were three‐ to five‐fold higher than that seen with the wild‐type MFα signal peptide (Fig. 2E and F). The constitutive promoter‐driven mutation‐accumulated strains showed trends of scFv production that were similar to those seen with the *AOX1* promoter‐driven constructs: the single‐amino‐acid substitution, 2xAdv, and 3xAdv strains showed relatively high levels of scFv production, while the 4xAdv and 8xAdv strains displayed scFv production levels comparable to (though nominally lower than) those of the single‐amino‐acid substitution strains. However, the αpp8 and αppS4 strains showed high scFv secretion levels for constructs driven by the *GAPDH* promoter (Fig. 2E and F), in contrast to the results seen with the *AOX1* promoter‐driven constructs (Fig. 2A, E and G). To assess whether these differences reflected the presence of methanol, we repeated the scFv antibody production assays with the constitutive *GAPDH* promoter‐driven strains grown in BMMY (methanol) medium (Fig. S2). Although these *GAPDH* promoter strains grown in the BMMY culture conditions showed lower protein secretion levels than those grown in the BMGY culture conditions (Fig. 2E and Fig. S2), the profiles of protein secretion were similar among the tested strains (Fig. 2H), indicating that the difference in carbon source was not responsible for the decrease in the scFv secretion levels seen with the *AOX1* promoter.

To evaluate the protein secretion ability of the yeast strains with the three distinct report gene‐swap constructs (all under control of the *AOX1* promoter), we determined protein production levels by measuring fluorescence (for GFP), reactivity with a chromogenic substrate (as assessed by absorbance at 405 nm, for BGL1p) and ELISA titre (for BiTE) of the culture supernatants. For the BGL1p and GFP constructs, because most enzymes and fluorescent proteins are known to have pH dependencies for their activities and their fluorescence (respectively), we first measured pH values of the culture supernatants at the end of the 48‐h BMMY cultivation, for each of the MFα mutation strains and the host strain. All secreting strains indicated the same pH value (approximately pH 6.3) as the host strain, except for slightly higher pH values (pH 6.5 ~ 6.6) in the 8xAdv and αpp8 control strains in the GFP secretion constructs (Fig. S3). Thus, the changes in pH appeared to be negligible for comparisons of protein secretion levels of the amino acid substitution strains using GFP fluorescence and BGL1p activity in the culture supernatants.

**Fig. 3:**
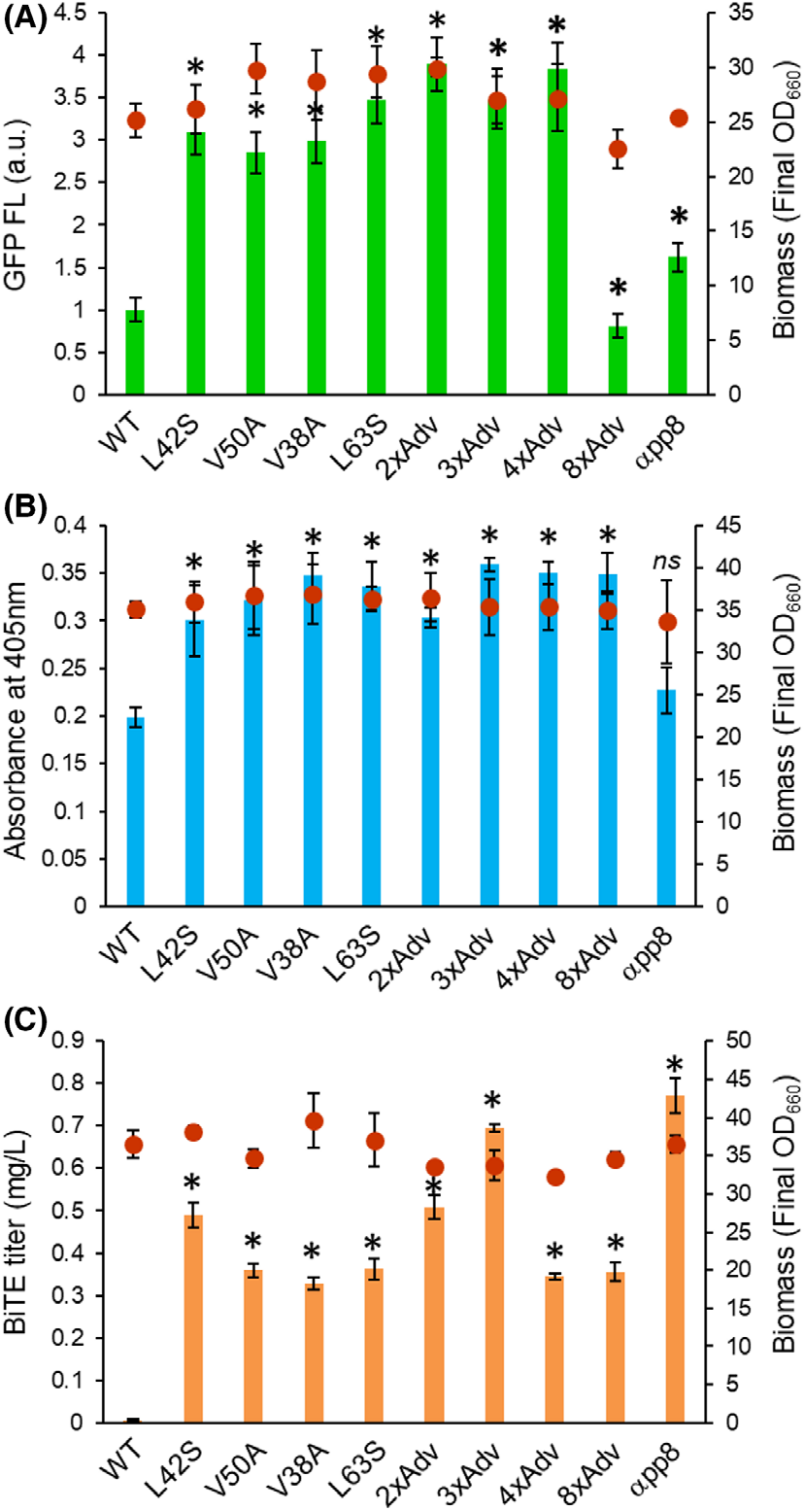
Target protein‐swap experiments (scFv to GFP, BGL1p, or BiTE). These plots show green fluorescence of mUkG1 (GFP) (A), absorbance derived from β‐glucosidase activity (BGL1p) (B), and titre of blinatumomab (BiTE) (C) from *P. pastoris* strains expressing these proteins with wild‐type and mutated MFα signal peptides. All of the MFα amino acid substitutions tested in Fig. 2A were tested here, except for αppS4 mutant. Bars and dots indicate protein production levels and final OD_660_ values, respectively. Values are plotted as the mean and standard deviation from three biological replicates. Asterisks indicate significance (*P* < 0.05 by two‐tailed non‐paired Student’s *t*‐test) for the comparison between strains encoding proteins with the wild‐type and mutant MFα signal peptides.

In the gene‐swap experiments using the two natural proteins (GFP and BGL1p), the single‐substitution and mutation‐accumulated strains exhibited 1.5‐ to 4‐fold higher protein secretion than the respective wild‐type MFα strain (Fig. 3A and B). All the effective mutation‐accumulated strains exhibited protein secretion levels comparable to those obtained with the single‐amino‐acid substitution strains, with the exception of the 8xAdv‐ and αpp8‐mutation bearing strains. For the GFP reporter strains, the 8xAdv strain showed a level of protein secretion that was relatively low and comparable to that obtained with the wild‐type MFα strain (Fig. 3A and Fig. S4a); for the BGL1p reporter strains, the 8xAdv strain displayed a protein secretion that was relatively high and comparable to that seen with other signal‐mutated strains (Fig. 3B and Fig. S4a). The αpp8 strain exhibited relatively low secretion levels both for GFP and BGL1p compared to other signal‐mutated strains (Fig. 3A and B, Fig. S4a). When the artificial protein (BiTE) was used as the reporter, much higher levels of protein secretion (50‐ to 80‐folds) were observed in the single‐amino‐acid mutated strains than in the wild‐type MFα strain (Fig. 3C and Fig. S4b). The effective mutation‐accumulated strains also yielded BiTE secretion titres comparable to those seen with the single‐amino‐acid substitution strains. In contrast, 3xAdv and αpp8 mutations resulted in significantly higher (100‐ to 120‐fold) levels of BiTE secretion.

Considered together, these data revealed that the effective single‐amino‐acid substitutions in the MFα signal peptide brought about significantly improved protein secretion in *P. pastoris* independent of carbon source (glucose and glycerol) in the culture medium, although there were some differences in the production levels and the effects depending on the promoters and the reporter proteins. Among the single‐amino‐acid substitutions in the MFα signal peptide that were tested here, only the L42S mutation’s effect (originally contained in Invitrogen’s pPICZα plasmid) has been described previously (Barrero *et al*., 2018). To our knowledge, the effects of the other of single‐amino‐acid mutations in the MFα signal have not previously been investigated alone. On the other hands, among the MFα signal peptide mutants with multiple‐amino‐acid substitutions, 8xAdv and αpp8 demonstrated protein production levels that were largely dependent on the promoters and the proteins, while 2xAdv (L42S/V50A) and 3xAdv (L42S/V50A/V38A) displayed relatively stable elevated production across the various tested reporter proteins. Notably, none of the combinatorial cumulative mutations provided apparent improvement of protein secretion levels compared to the single‐amino‐acid substitutions.

### Changes in hydrophobicity of single‐amino‐acid substitutions correlate with protein production

As demonstrated above, single‐amino‐acid substitutions in the MFα signal peptide led to improved protein secretion in *P. pastoris*, a pattern that applied across four different reporter proteins and two distinct promoter systems. These results raise the question of how these substitutions impact the protein secretion process in *P*. *pastoris*. To address this question, we next investigated the mechanism(s) underlying effects seen with single‐amino‐acid substitutions in the MFα signal peptide. This work employed the scFv‐encoding constructs expressed under the control of the *AOX1* promoter.

**Fig. 4:**
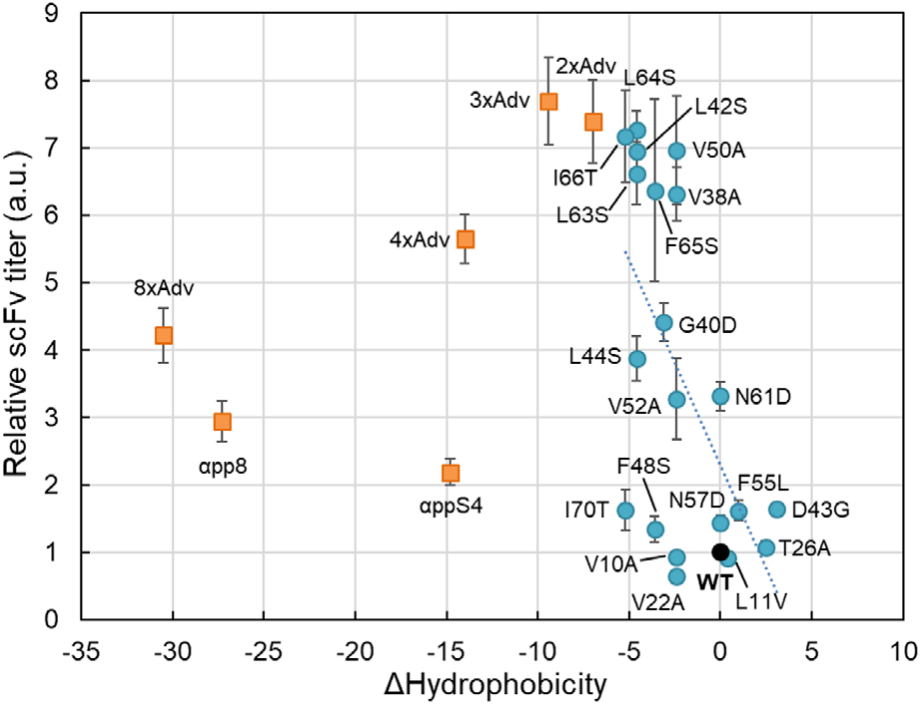
Plots of change in hydrophobicity of peptides harbouring the amino acid substitutions versus the respective scFv titres. The scFv titre data of strains encoding scFv with the wild‐type (black circle) signal peptide, with each of the 20 single (blue circles) and six multiple‐amino‐acid substitutions in the signal peptide (orange rectangles) were as in Fig. 1B and as in Fig. 2A respectively. The hydrophobicity index was as described in a previous report (Kyte and Doolittle, 1982). The ∆hydrophobicity was calculated as the difference in hydrophobicity value between the original and substituted amino acid(s). The correlation between the change in hydrophobicity and the scFv titre for each single‐amino‐acid substitution indicated a statistically significant correlation with Pearson’s *r* (−0.599) and *P* value (4.1 × 10^−3^).

To investigate the properties of single‐amino‐acid substitutions for obtaining the higher scFv titres, we first assessed the correlation between the scFv titres and the changes in hydrophobicity imparted by each of the amino acid substitutions because of a success of secretion improvement for changing more polar residues at the native motif in the MFα signal peptides (Rakestraw *et al*., 2009). Using a hydrophobicity index of amino acids (Kyte and Doolittle, 1982), changes in hydrophobicity were calculated for MFα signal peptides harbouring each of the twenty single‐amino‐acid substitutions (Fig. 1A, orange and red). The changes in hydrophobicity correlated significantly with the scFv titre values derived from the 20 single‐amino‐acid substitution strains (Pearson’s *r* = −0.599, *P* = 4.1 × 10^−3^; Fig. 4). Thus, it appears that each of the seven MFα signal peptide substitutions that elevate the titres of secreted protein also lower the hydrophobicity of the signal peptide (Fig. 4).

To identify other physicochemical and biological properties of the single‐amino‐acid changes that may correlate with the scFv titres, we used an amino acid index database, AAindex (Kawashima *et al*., 2008) which had the 566 indices including hydrophobicity, polarity, pKa, pI, net charge, diameter, molecular weight and so on, and searched for parameters that showed the strongest fit to reporter protein levels by using a correlation analysis (see Experimental procedure section). However, compared to the hydrophobicity index (Kyte and Doolittle, 1982), the other indices in the AAindex showed comparable or lower correlation with scFv titres than did hydrophobicity (Table S1), indicating that hydrophobicity is one of the most useful properties among known amino acid characteristics for explaining the observed changes in scFv titres in *P. pastoris*.

Subsequently, among the MFα signal peptide mutants with the multiple‐amino‐acid substitutions, we also compared the changes in hydrophobicity of the amino‐acid substitutions with the scFv titres. The results showed that the 2xAdv and 3xAdv positioned on the correlation between the change in hydrophobicity and the scFv titres for the single‐amino‐acid substitutions described above, while the large mutation accumulations in the MFα signal peptide having much lower change in hydrophobicity (less than −10) were left out of the correlation (Fig. 4). Together, hydrophobicity was shown to be one index to relate to the scFv titre in a small range around the value of the wild‐type MFα signal peptide, but cannot explain it with that alone. There will be another mechanism to be explored.

### UPR induction and GFP localization analyses reveal that avoiding entry into protein degradation pathways is important for improving protein secretion

We next checked whether the unfolded protein response (UPR) was activated by accumulation of unfolded proteins in the ER following induction of the expression of secreted GFP with wild‐type and mutated MFα signal peptides. Previous work evaluated the degree of the UPR using a UPR biosensor that assessed the fluorescence intensity of yeast cells carrying a construct expressing a fluorescent protein‐encoding gene under the control of the *KAR2* promoter (Lajoie *et al*., 2012; Cedras *et al*., 2020). We constructed a UPR biosensor strain that carries a red fluorescent protein (RFP) E2Crimson‐encoding gene downstream of the *KAR2* promoter, and then introduced into this strain constructs encoding secreted GFP fused to either wild‐type or mutated MFα signal peptides. After 48 h of cultivation in BMMY, fluorescence of GFP (in the culture supernatant) and RFP (in the yeast cells) were measured with a microplate reader and a flow cytometer, respectively.

**Fig. 5:**
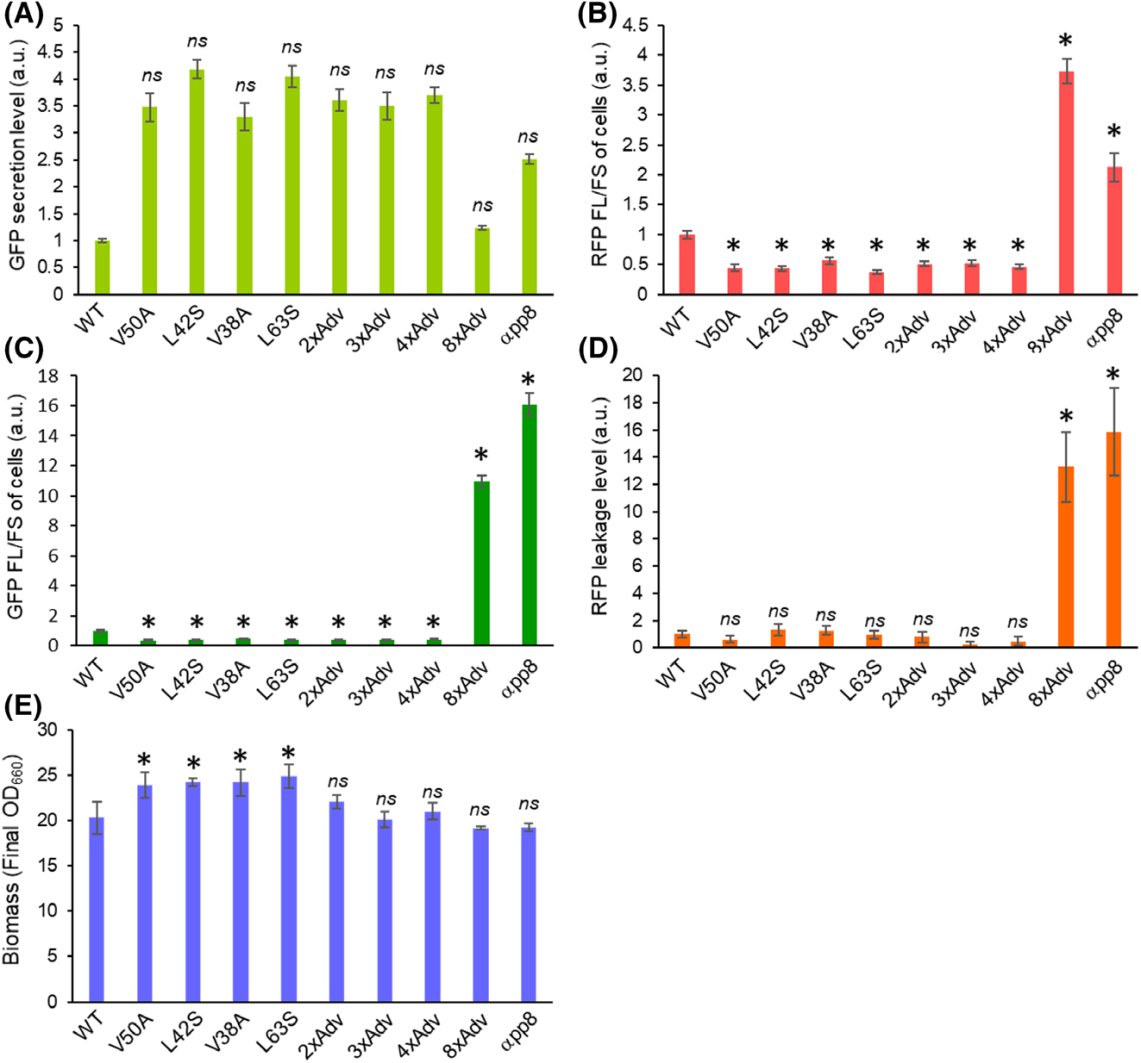
UPR biosensor analysis of strains secreting GFP expressed with various MFα signal peptides. GFP production (A) and RFP leakage (D) were evaluated by fluorescence, as assessed using a microplate reader. The remaining GFP (C) and RFP fluorescence (UPR activation) levels (B) were measured by fluorescence in yeast cells, as assessed by flow cytometry. (E) Biomass (final OD_660_) values of each strain following cultivation in BMMY (methanol). Values are plotted as the mean and standard deviation from three biological replicates. Asterisks indicate significance (*P* < 0.05 by two‐tailed non‐paired Student’s *t*‐test) for the comparison between strains encoding proteins with the wild‐type and mutant MFα signal peptides.

The GFP fluorescence for culture supernatants of the UPR‐evaluating strains (carrying the *KAR2* promoter‐E2Crimson construct) indicated trends that resembled those of the original GFP secretion strains (i.e. without the UPR biosensor construct; Figs 3A and 5A). The GFP fluorescence readouts of the supernatant (the GFP secretion level) for the four tested single‐amino‐acid substitution strains were considerably (3‐ to 4‐fold) higher than that of the wild‐type MFα strain (Fig. 5A). As expected, cells of these four single‐amino‐acid substitution strains showed low levels of RFP fluorescence (RFP FL/FS), indicating that the UPR levels in these strains might be at ground state (Fig. 5B). In contrast, the wild‐type MFα signal strain displayed approximately two‐folds higher RFP fluorescence of the cells than the single substitution strains with significant difference (*P* < 0.05) (Fig. 5B), suggesting that the UPR was slightly activated in ER of the wild‐type MFα strain. The 2xAdv, 3xAdv, and 4xAdv strains, which exhibited high levels of secreted GFP production (Fig. 5A), showed cellular RFP fluorescence comparable to that of the single‐amino‐acid substitution strains (Fig. 5B). The 8xAdv and αpp8 strains, for which GFP secretion was relatively low (Fig. 5A), showed RFP cellular fluorescence that was seven‐fold and four‐fold higher than that seen in the other strains with significant difference (*P* < 0.05), respectively (Fig. 5B and Fig. S5a). These results indicated that single‐amino‐acid substitution and modest accumulation of effective mutations in the MFα signal peptide enhance protein secretion, but accumulation of such mutations may reduce the secreted protein production due to strong UPR induction.

Subsequently, we focused on GFP fluorescence that was retained in the yeast cells (GFP FL/FS of cells) and RFP leakage into the culture supernatant (RFP leakage level) for these GFP‐secreting strains following cultivation in BMMY. The wild‐type MFα strain, which had mild UPR activation (as shown above), exhibited a slight but statistically significant (*P* < 0.05) elevation in cellular GFP fluorescence (Fig. 5C). Strains (8xAdv and αpp8) that were strongly induced for the UPR exhibited not only elevated cellular GFP fluorescence (Fig. 5C and Fig. S5b) but also increased leakages of RFP into the culture supernatant (Fig. 5D and Fig. S5c). We hypothesized that these effects reflected cell damage resulting from ER stress arising from unprocessed (non‐secreted) GFP. The biomass (final OD of yeast culture) of the single‐amino‐acid substitution strains showed significantly higher than that of the wild‐type MFα strain, resulting from the low (almost basal) UPR levels (Fig. 5E).

**Fig. 6:**
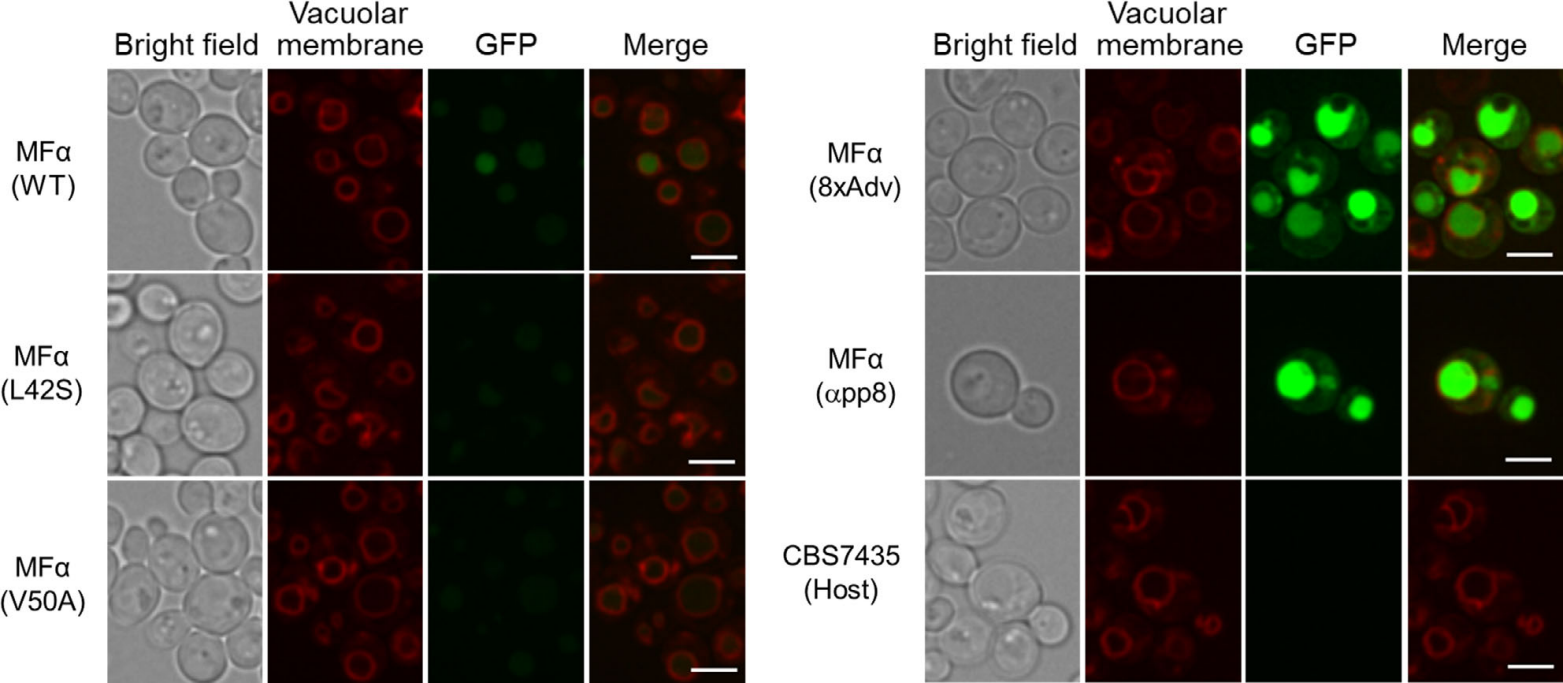
Localization analysis of GFP in *Pichia pastoris* cells. Fluorescence images of the GFP‐secreting strains with MFα wild‐type, single‐ and multiple‐amino‐acid substitutions (L42S, V50A and 8xAdv mutations) and αpp8 mutant under the control of the methanol‐inducible *AOX1* promoter. The cells were cultured in BMMY (methanol) medium for 48 h, stained with FM 4‐64 (red) to visualize vacuolar membranes, washed, and then observed using a confocal microscope. The MFα wild‐type strain showed slight GFP fluorescence in vacuoles in the yeast cells. Scale bars, 5 μm.

As mentioned above, the wild‐type MFα strain retained a slightly increased level of cellular GFP fluorescence compared to the single‐amino‐acid substitution strains (Fig. 5C). To visualize where the retained green fluorescence was located within the yeast cells, we performed fluorescence microscopy on two single‐amino‐acid substitution strains (V50A and L42S), as well as on the wild‐type MFα signal peptide strain and the host strain. Here, the original GFP secretion strains (without the UPR biosensor) were used to permit (in the absence of RFP) staining of the vacuolar membrane with a red fluorescent dye, FM 4‐64 (Vida and Emr, 1995; Heiss *et al*., 2015). As expected, cellular GFP fluorescence was effectively absent from either of the single‐amino‐acid substitution strains (V50A and L42S) as well as from the host strain (CBS7435) (Fig. 6). Interestingly, the wild‐type MFα strain showed a slight cellular GFP fluorescence; notably, this fluorescence localized to vacuoles (Fig. 6), suggesting that the single‐amino‐acid substitutions prevent the mis‐sorting of the recombinant proteins into the degradation pathway in vacuoles. On the other hand, the strains (8xAdv and αpp8) that exhibited strong induction of UPR showed abnormal GFP fluorescence within cells; again, GFP in these cells accumulated in the vacuoles (Fig. 6).

The analysis of the UPR activation showed that the single substitution or modest mutation‐accumulated MFα signal peptide strains did not induce UPR activation, while the wild‐type MFα signal peptide strains exhibited slightly induction of UPR above basal levels. These results suggested that the pro‐region of the wild‐type MFα signal peptide may be prone to aggregation in the ER, consistent with previous work (Barrero *et al*., 2018). Moreover, slight accumulation of secreted protein (GFP) in vacuoles was observed for the UPR‐induced strain encoding the wild‐type MFα signal peptide, consistent with previously reported results (Kunze *et al*., 1999; Fitzgerald and Glick, 2014). In contrast, the accumulation of GFP in vacuoles was not observed for the UPR‐non‐induced single‐amino‐acid substitution strains (L42S and V50A). Thus, the wild‐type MFα signal apparently induces, to a low level, both of two major intracellular protein degradation pathways in yeast, including ER‐associated degradation (ERAD; one of the destinations of proteins in UPR‐activated cells) and a mis‐sorting of the recombinant proteins into vacuoles (Idiris *et al*., 2010; Puxbaum *et al*., 2015). Strains encoding reporter proteins with the single effective mutations presumably reduce these activities, which are counter‐productive for secretion of recombinant proteins. In contrast, strains encoding multiply mutated signal peptides (8xAdv and αpp8) strongly activated the UPR and accumulated much higher levels of recombinant secretory protein in the vacuoles. These differences presumably are attributable to the large decreases in signal peptide hydrophobicity resulting from multiple signal peptide mutations, which in turn strongly induce UPR activation and mis‐sorting to vacuoles, resulting in an overload of the protein degradation systems.

In the present study, we showed that the various single‐amino‐acid substitutions in the MFα signal peptide are highly effective for enhancing the secretion of recombinant proteins in *P. pastoris*. We also provided preliminary experimental evidence for the mechanism(s) underlying the role of the effective amino acid substitutions in the MFα signal peptide: decreasing UPR activation and avoiding the entry of recombinant proteins into intracellular protein degradation pathways during the process of secretion. These results are expected to facilitate the further engineering of high‐producer strains for recombinant protein secretion in *P. pastoris*.

## Experimental procedures

### Strains and media conditions

*Escherichia coli* strain DH5α was used for recombinant DNA manipulation. *E. coli* strains were grown in LB medium as described in our previous paper (Ito *et al*., Submitted).

*Pichia pastoris* wild‐type strain CBS7435 (NRRL‐Y11430) was used in this study. The yeast strains were grown in YPD, or in BMGY or BMMY media [10 g l^−1^ yeast extract, 20 g l^−1^ hipolypeptone (Nihon Pharmaceutical, Tokyo, Japan), 13.4 g l^−1^ yeast nitrogen base without amino acids (BD Biosciences, San Jose, CA, USA), 0.4 mg l^−1^ biotin (Nacalai Tesque, Kyoto, Japan), 100 mM potassium phosphate buffer (final, pH 6.0) and 20 g l^−1^ glycerol (for BMGY) or 20 g l^−1^ methanol (for BMMY)], as described in our previous paper (Ito *et al*., 2020). Rich medium consisted of YPD agar plates containing YPD broth, 20 g l^−1^ agar, and 500 μg ml^−1^ G418 (FUJIFILM Wako Pure Chemical, Osaka, Japan).

### Construction of plasmids and *P. pastoris* strains

Standard recombinant DNA manipulations were as described by Sambrook and Russel (2001). The plasmids, primers and synthetic DNAs used in this study are listed in Tables S2–S4 respectively. Primers were purchased from Eurofins Genomics K.K. (Tokyo, Japan); synthetic DNA fragments were synthesized by GeneArt Strings DNA Fragments (Thermo Fisher Scientific, Waltham, MA, USA).

The plasmids used for *AOX1* and *GAPDH* promoter‐driven expression and secretion of anti‐lysozyme scFv(H‐L) (pPAP_scFv and pPGP_scFv respectively) and the plasmid used for *AOX1* promoter‐driven expression and secretion of BGL1p (pPAP_BGL1) were described in our previous study (Ito *et al*., Submitted). These three plasmids harbour sequences encoding the wild‐type MFα signal peptide, positioned downstream of the promoters. pPAP_mUkG1 and pPAP_Blin, the plasmids used for *AOX1* promoter‐driven expression and secretion of mUkG1 (GFP) (Tsutsui *et al*., 2008; Kaishima *et al*., 2016) and of a bispecific T‐cell engineering antibody, blinatumomab (BiTE) (Löffler *et al*., 2000) respectively, were constructed as part of the present work. Specifically, the open reading frame (ORF) of pPAP_scFv that encodes scFv was replaced with codon‐optimized (for expression in *P. pastoris*) synthetic DNA fragments encoding mUkG1 or BiTE. ORF replacement was performed using In‐Fusion technology (Takara Bio, Shiga, Japan).

Introduction of mutations coding for single‐ and multiple‐amino‐acid substitutions in the MFα signal peptide was performed on the plasmid pPAP_scFv by use of an inverse‐PCR method. The inverse‐PCR was performed with primers carrying the mutation(s) of interest, followed by treatment with polynucleotide kinase, self‐ligation, and recovery by transformation into *E. coli* DH5α. Plasmids encoding the previously reported (control) MFα signals (αpp8 and αppS4) (Rakestraw *et al*., 2009) were constructed by replacing (again, using In‐Fusion technology) pPAP_scFv sequences encoding wild‐type MFα signal peptide with synthetic DNA fragments encoding the mutated MFα signal peptides. The DNA fragments encoding the MFα signal peptide mutants were transferred into the plasmids pPAP_mUkG1, pPAP_BGL1, pPAP_Blin, and pPGP_scFv (which expresses scFv under control of the *GAPDH* promoter) by cloning via SpeI‐, XhoI‐ended fragments. The identities of all the plasmids were confirmed by DNA sequencing.

These integrative plasmids (pPAP_scFv, pPGP_scFv, pPAP_mUkG1 and pPAP_Blin) encoding reporter proteins with wild‐type and mutant MFα signal peptides were transformed into yeast. In brief, plasmids were linearized with EcoRV and then introduced into *P. pastoris* CBS73435 strain by the lithium acetate method, resulting in integration into the terminator region of the chromosomal *CCA38473* locus, as described in our previous paper (Fig. S6) (Ito *et al*., 2018).

The UPR biosensor plasmid pPKP_E2Crimson was constructed as follows. The plasmid pUC19‐MCS‐Zeo, which carries a multiple cloning site (MCS) linker (HindIII‐PstI‐BamHI‐SpeI‐XhoI‐BglII‐EcoRI), was constructed by insertion of a synthetic DNA fragment, a zeocin‐resistance‐encoding cassette, and a MCS sequence into the HindIII/EcoRI site of pUC19 (Takara Bio). DNA fragments corresponding to the *P. pastoris ARG4* gene, the *KAR2* promoter, and *AOX1* terminator were amplified by PCR using *P. pastoris* genomic DNA as a template and appropriate primers. Separately, DNA fragments corresponding to a E2Crimson (RFP) (Strack *et al*., 2009)‐encoding ORF were synthesized. The resulting DNA fragments corresponding to the *ARG4* locus, *KAR2* promoter, the RFP‐encoding ORF and *AOX1* terminator were inserted sequentially into the HindIII/BamHI, BamHI/SpeI, SpeI/XhoI, XhoI/BglII, and EcoRI sites of pUC19, respectively. The resulting plasmid, pPKP_E2Crimson, expresses RFP under the control of the constitutive *KAR2* promoter. The identity of this plasmid was confirmed by DNA sequencing.

### Cultivation of yeast strains

Test tube cultivation was performed for the quantification of the protein secretion levels of the MFα signal peptide mutant strains in all experiments in this study. At least three biologically distinct colonies were streaked on YPD plates supplemented with 500 μg ml^−1^ G418 for each *P. pastoris* transformant; each then was inoculated into 2 ml of BMGY medium in a test tube and incubated (pre‐cultured) with shaking (170 rpm) at 30°C for 24 h. An aliquot (200 µl) of each pre‐culture was used to inoculate 2 ml of BMMY (for the *AOX1* promoter strains) or BMGY medium (for the *GAPDH* promoter strains) in a test tube, and additionally incubated (for protein secretion) with shaking (170 rpm) at 30°C for 48 h. After cultivation, the biomass of this culture was assessed by optical density at 660 nm (OD_660_), and the protein secretion level of the reporter protein was analysed as described below.

### Construction of yeast strain library carrying MFα signal peptide mutations

A MFα mutation library with various combinations of the seven amino acid substitutions (V38A, L42S, V50A, L63S, L64S, L65S and I66T) was generated as follows. Degenerate primers encoding mutations (Y or R) at the corresponding seven sites on the MFα signal peptide (Table S3) were used for inverse‐PCR amplification of the plasmid pPAP_scFv; the resulting products were treated with polynucleotide kinase and self‐ligated. The resulting plasmid library was electroporated into HST08 competent cells (Takara Bio) and transformants were plated on LB plates supplemented with 100 μg ml^−1^ ampicillin (LB Amp). The resulting transformants (around 10 000 colonies) on a given LB Amp plate were pooled, and a plasmid library (MFα signal library) was extracted by QIAGEN Plasmid *Plus* Midi kit (Qiagen, Venlo, Netherlands). To prepare the yeast MFα signal library, the MFα signal plasmid library was linearized and then electroporated into the *P. pastoris* CBS7435 strain. The scFv secretion abilities of the yeast transformants were evaluated using the high‐throughput screening system described previously (Ito *et al*., Submitted).

### ELISA for simple quantification of small antibody secretion titre

The titres of the secreted scFv and BiTE were determined from the 48‐h culture supernatants using a sandwich enzyme‐linked immunosorbent assay (ELISA) assay, conducted essentially as described in our previous report (Ito *et al*., Submitted), except for the BiTE detection assay. For the titre evaluation of BiTE, 4 ng ml^−1^ Protein L (ProSpec, Rehovot, Israel) was used to coat the ELISA plate, and horseradish peroxidase (HRP)‐conjugated chicken anti‐6x His tag antibody (Abcam, Cambridge, UK) was used to detect BiTE, which had a His_6_‐tag.

### Western blot analysis

The amount of scFv antibody secreted from each yeast strain was evaluated by a western blot analysis. Consistent volumes of BMMY culture supernatants obtained at the final culture point (48 h) for each yeast strain were separated by sodium dodecyl sulphide‐polyacrylamide gel electrophoresis (SDS‐PAGE). Proteins were transferred to a nitrocellulose membrane using a blotting system (iBlot Gel Transfer System, Thermo Fisher Scientific) according to the manufacturer’s instructions, and non‐specific binding was blocked by incubating the membrane in a blocking reagent (BlockingOne, Nacalai Tesque) at room temperature for 1 h. The blocked membrane then was incubated at room temperature for 1 h with rabbit anti‐6‐His Tag antibody (Bethyl Laboratories, Montgomery, TX, USA) diluted 1:5000 in TBS‐T buffer (10 mM Tris‐HCl, pH 8.0, 150 mM NaCl, 0.05% Tween20). After washing the membrane with TBS‐T buffer, scFv was detected by incubation at room temperature for 1 h in alkaline phosphatase (AP)‐conjugated anti‐rabbit IgG (Fc) (Promega, Madison, WI, USA) diluted 1:500 in TBS‐T buffer. After again washing the membrane with TBS‐T buffer, the antibody‐antigen complex was detected by addition of a luminescent substrate (CDP‐STAR Detection Reagent, GE Healthcare, Little Chalfont, UK) and visualized using a charge‐coupled device (CCD) imager (LAS‐4000, GE Healthcare).

### Growth curve analysis

Growth rates of the gene‐disrupted strains were monitored by tracking of OD_660_ values during the exponential growth phase, as described previously (Ito *et al*., 2018). Briefly, the yeast strains were streaked on YPD plates supplemented with G418 and incubated at 30°C for 2 days. Individual clones then were used to inoculate test tubes containing BMGY. After overnight incubation with shaking at 30°C, each culture was diluted to an initial OD_660_ of 0.05 in L‐shape tubes containing BMMY. The diluted cells then were cultured at 30°C using a Bio‐photorecorder (TVS062CA; Advantech, Tokyo, Japan), and the OD_660_ values were measured at 30‐min intervals. Three biological replicates were tested for each strain.

### RT‐qPCR

The mRNA level of each signal mutated yeast strain was measured by reverse transcription‐quantitative PCR (RT‐qPCR), as described previously (Ito *et al*., 2020). The levels of the mRNA encoding scFv antibody with wild‐type or mutant signal peptide were determined after 6 h of cultivation in BMMY (methanol) medium.

### Evaluation of BGL1p, GFP secretion and RFP leakage levels

The levels of secreted BGL1p and GFP (and of leaking RFP, a marker of cell damage in the UPR detection experiment described below) were evaluated by assessing BGL1p activity or GFP fluorescence (respectively) of the yeast culture supernatant after cultivation for 48 h in BMMY medium. BGL1p activity was assayed as described previously (Inokuma *et al*., 2016). GFP secretion and RFP leakage were assayed as described below. Transformants secreting mUkG1 were cultured in test tubes as described above. Aliquots (150 µl each) of the supernatant of each test tube culture were transferred to a 96‐well black plate (Greiner Bio‐One, Frickenhausen, Germany) and monitored for GFP fluorescence (excitation: 485/14 nm, emission: 535/25 nm) and RFP fluorescence (excitation: 611 nm, emission: 646 nm in the monochromator mode) using a microplate reader (Envision; Perkin Elmer, Waltham, MA, USA) to measure the levels of GFP secretion and RFP leakage (respectively).

### Flow cytometry

To determine the levels of intracellular fluorescence, transformants producing GFP and RFP were cultured in test tubes as described above. Fluorescent intensities of 10 000 cells of each strain were measured using a flow cytometer (CytoFlex; Beckman Coulter, Brea, CA, USA) equipped with 488‐ and 638‐nm laser and the appropriate filter sets for GFP (525/40 nm) and RFP (675/30 nm). Data processing was performed such that the median value of the fluorescent intensity for GFP or RFP was divided by the median value of the forward scatter intensity to compensate for any differences in cell size. The degree of the UPR induction and the amount of GFP remaining in yeast cells (not yet secreted) were compared among the MFα signal peptide mutant strains.

### Fluorescence microscopy

GFP‐secreting transformants were cultured in test tubes as described above. After cultivation for 48 h in BMMY medium, vacuoles in the cells were stained using FM 4‐64 (Vida and Emr, 1995; Heiss *et al*., 2015). Briefly, the cells were incubated in the dark at 30°C for 15 min with shaking in the presence of 15 μM FM 4‐64 (Invitrogen, Carlsbad, CA, USA) diluted in BMMY medium. The cells then were washed and resuspended in BMMY medium, followed by an additional 1 h incubation in the dark at 30°C with shaking. After being washed twice with phosphate‐buffered saline (PBS) to remove any secreted GFP and unattached dye, the cells were observed using a BZ‐X810 fluorescence microscope equipped with a sectioning function (Keyence, Osaka, Japan), a Nikon Plan Apo Lambda 100x/1.45 oil‐immersion objective lens (Nikon, Tokyo, Japan), and appropriate filters for GFP and FM 4‐64.

### Correlation analysis using AAindex

We used the amino acid index (AAindex) database Release 9.2 (Kawashima *et al*., 2008) (https://www.genome.jp/aaindex) to quantify the properties of the wild‐type signal peptide sequence and 20 single‐amino‐acid mutant sequences; we then calculated the correlation coefficient between these properties and scFv titres. Specifically, for each mutant sequence and for each index, we calculated the difference in properties between the wild‐type and the mutant sequence as follows.

$$d=I\left(k,\;aa_{m}\right)‐I\left(k,aa_{w}\right),$$

where *aa_m_* and *aa_w_* are the mutated amino acid in the mutant sequence and the original amino acid in the mutated position respectively. I(*k*, *aa_m_*) and I(*k*, *aa_w_*) are the index values of *aa_m_* and *aa_w_* in the *k*th index respectively. For each of the 566 indices, we obtained the *d* values for each of the 20 mutant sequences. The *d* value of the wild‐type sequence was set to 0. We then calculated the Pearson’s correlation coefficient between the *d* values and the scFv titres. All indices were ranked according to the absolute value of the correlation coefficient.

### Statistical tests

Two‐tailed non‐paired Student’s *t*‐tests were used to determine the significance of the differences between groups of values. *P* values less than 0.05 were considered to be statistically significant.

## Conflict of interest

None declared.

## Supporting information


**Fig. S1**. Screening of a library of constructs encoding random combinations of MFα signal mutations. The levels of secreted scFv generated by each of approximately 900 yeast strains (corresponding to ten 96‐well deep‐well plates used in this screen) with various combinations of the top‐seven‐ranked effective single amino acid substitutions (V38A/L42S/V50A/L63S/L64S/F65S/I66T; diversity: 2^7=128) are indicated as raw ELISA data. Red lines indicate the level of expression by constructs encoding proteins with the MFα V50A mutation, which served as a control and was positioned in a single well in each of the ten 96‐well deep‐well plates screened in this assay.
**Fig. S2**. Promoter‐swap experiment (*AOX1* to *GAPDH* promoter). This figure shows properties of *Pichia pastoris* strains expressing secreted scFv under the control of the constitutive *GAPDH* promoter. The amino acid substitution mutants used were as in Figure 2a. The scFv titres were those of the strains encoding proteins with various MFα mutations following culture in BMMY (methanol). scFv titres (bars) and biomass (final OD_660_) (circles) are indicated. Values are plotted as the mean and standard deviation from three biological replicates. Asterisks indicate significance (*p* < 0.05 by two‐tailed non‐paired Student’s *t*‐test) for the comparison between strains encoding proteins with the wild‐type and mutant MFα signal peptides.
**Fig. S3**. pH of the culture supernatants. pH values of culture supernatants were measured for *Pichia pastoris* strains secreting GFP (a) and BGL1p (b) that were expressed with wild‐type (WT) and various MFα signal peptide mutants harbouring amino acid substitutions. pH of the supernatant of the host CBS7435 strain also was measured as a control. The GFP‐ and BGL1p‐secreting strains used were the same as those employed in Figure 3a and 3b. These strains were cultured in BMMY (methanol) for 48 h. Values are plotted as the mean and standard deviation from three biological replicates. Asterisks indicate significance (*p* < 0.05 by two‐tailed non‐paired Student’s *t*‐test) for the comparison between strains encoding proteins with the wild‐type and mutant MFα signal peptides.
**Fig. S4**. Reporter gene‐swap experiments. Relative titres (compared to the wild‐type MFα signal peptide strains) for β‐glucosidase (BGL1p) versus mUkG1 (GFP) strains (a) and BGL1p versus blinatumomab (BiTE) (b) are indicated. All strains with MFα signal peptides containing amino acid substitutions were as used in Fig. 2a, except that the αppS4 mutant was not tested. Values are plotted as mean ± standard deviation of three biological replicates.
**Fig. S5**. UPR biosensor analysis of mUkG1 (GFP)‐secreting strains with single and combined amino acid substitutions in the MFα signal peptide. Cells were cultured in BMMY (methanol) medium to induce the GFP expression under the control of *AOX1* promoter. A *KAR2* promoter‐*E2Crimson* (RFP)‐*AOX1* terminator construct was introduced into the GFP‐secreting strains. All of the MFα signal peptide amino acid substitutions were as used in Fig. 2a, except that the αppS4 mutant was not tested. The GFP and RFP fluorescence levels of the culture supernatant and the cells were measured using a microplate reader and a flow cytometer, respectively. GFP secretion (GFP fluorescence of culture supernatant) (a), remaining GFP in cells (intracellular GFP fluorescence) (b) and RFP leakage (RFP fluorescence of culture supernatant) (c) were plotted against the UPR activation levels (intracellular RFP fluorescence).
**Fig. S6**. Schematic of genome integration for construction of *Pichia pastoris* strains. Sequences encoding wild‐type and mutated MFα signals were inserted in plasmids as modules downstream of a promoter (*GAPDH* or *AOX1* promoter) and upstream of a gene of interest (GOI). In various experiments, the GOI encoded anti‐lysozyme scFv, β‐glucosidase (BGL1p), mUkG1p (GFP) or blinatumomab bispecific antibody (BiTE). Following linearization of the plasmid with EcoRV (or NruI) and transformation into CBS7435 (*P. pastoris* wild‐type strain), homologous integration occurred in the genomic copy of the *CCA38473* (*T38473*) terminator region. A G418 resistance‐encoding marker was used for selection. Grey arrows indicate positions of the primers used in colony PCR to confirm correct integration events.

**Table S1**. Amino acids indices correlated with the scFv titre values.
**Table S2**. Characteristics of plasmids used in this study.
**Table S3**. Primers used in this study.
**Table S4**. Synthetic DNAs used in this study.
