# Supplementary material for: Avoiding entry into intracellular protein degradation pathways by signal mutations increases protein secretion in Pichia pastoris
Source: Microb Biotechnol. 2022 Jun 3;15(9):2364–78. doi: 10.1111/1751-7915.14061 (PMC9437885; doi:10.1111/1751-7915.14061)
Supplement: Supplementary file 1 — Fig. S1. Screening of a library of constructs encoding random combinations of MFα signal mutations. The levels of secreted scFv generated by each of approximately 900 yeast strains (corresponding to ten 96‐well deep‐well plates used in this screen) with various combinations of the top‐seven‐ranked effective single amino acid substitutions (V38A/L42S/V50A/L63S/L64S/F65S/I66T; diversity: 2^7=128) are indicated as raw ELISA data. Red lines indicate the level of expression by constructs encoding proteins with the MFα V50A mutation, which served as a control and was positioned in a single well in each of the ten 96‐well deep‐well plates screened in this assay. Fig. S2. Promoter‐swap experiment (AOX1 to GAPDH promoter). This figure shows properties of Pichia pastoris strains expressing secreted scFv under the control of the constitutive GAPDH promoter. The amino acid substitution mutants used were as in Figure 2a. The scFv titres were those of the strains encoding proteins with various MFα mutations following culture in BMMY (methanol). scFv titres (bars) and biomass (final OD660) (circles) are indicated. Values are plotted as the mean and standard deviation from three biological replicates. Asterisks indicate significance (p < 0.05 by two‐tailed non‐paired Student’s t‐test) for the comparison between strains encoding proteins with the wild‐type and mutant MFα signal peptides. Fig. S3. pH of the culture supernatants. pH values of culture supernatants were measured for Pichia pastoris strains secreting GFP (a) and BGL1p (b) that were expressed with wild‐type (WT) and various MFα signal peptide mutants harbouring amino acid substitutions. pH of the supernatant of the host CBS7435 strain also was measured as a control. The GFP‐ and BGL1p‐secreting strains used were the same as those employed in Figure 3a and 3b. These strains were cultured in BMMY (methanol) for 48 h. Values are plotted as the mean and standard deviation from three biological replicates. Asterisks indicate s [file MBT2-15-2364-s002.docx]

**Supporting Information**

**Avoiding entry into intracellular protein degradation pathways by signal mutations increases protein secretion in *Pichia pastoris***

**Yoichiro Ito^a,b^, Misa Ishigami^c^, Noriko Hashiba^c^, Yasuyuki Nakamura^a,b^, Goro Terai^d^, Tomohisa Hasunuma^a,b^, Jun Ishii^a,b*^ and Akihiko Kondo^a,b,e,f*^**

^a^ Engineering Biology Research Center, Kobe University, Kobe, Japan

^b^ Graduate School of Science, Technology and Innovation, Kobe University, Kobe, Japan

^c^ Technology Research Association of Highly Efficient Gene Design (TRAHED), Kobe, Japan

^d^ Department of Computational Biology and Medical Sciences, Graduate School of Frontier Sciences, The University of Tokyo, Chiba, Japan

^e^ Department of Chemical Science and Engineering, Graduate School of Engineering, Kobe University, Kobe, Japan

^f^ Center for Sustainable Resource Science, RIKEN, Yokohama, Japan

**^*^Corresponding author:** Jun Ishii

Engineering Biology Research Center, Kobe University, 1-1 Rokkodai, Nada, Kobe 657-8501, Japan

Tel: +81-78-803-6356; Fax: +81-78-803-6192; E-mail: junjun@port.kobe-u.ac.jp

**^*^Corresponding author:** Akihiko Kondo

Graduate School of Science, Technology and Innovation, Kobe University, 1-1 Rokkodai, Nada, Kobe 657-8501, Japan

Tel: +81-78-803-6196; Fax: +81-78-803-6196; E-mail: akondo@kobe-u.ac.jp


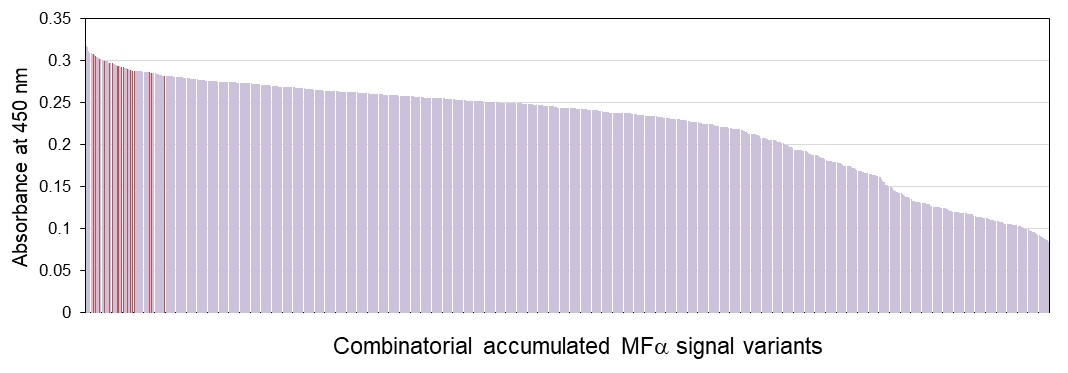


**Fig. S1 | Screening of a library of constructs encoding random combinations of MFα signal mutations**

The levels of secreted scFv generated by each of approximately 900 yeast strains (corresponding to ten 96-well deep-well plates used in this screen) with various combinations of the top-seven-ranked effective single amino acid substitutions (V38A/L42S/V50A/L63S/L64S/F65S/I66T; diversity: 2^7=128) are indicated as raw ELISA data. Red lines indicate the level of expression by constructs encoding proteins with the MFα V50A mutation, which served as a control and was positioned in a single well in each of the ten 96-well deep-well plates screened in this assay.


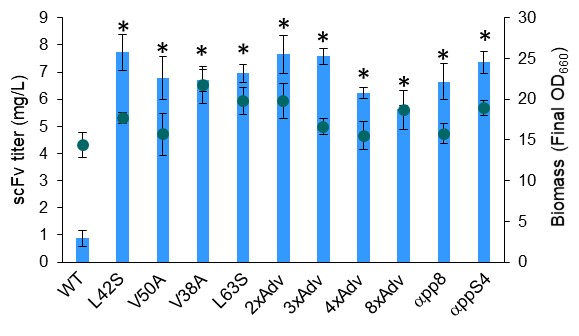


**Fig. S2 | Promoter-swap experiment (*AOX1* to *GAPDH* promoter)**

This figure shows properties of *Pichia pastoris* strains expressing secreted scFv under the control of the constitutive *GAPDH* promoter. The amino acid substitution mutants used were as in Figure 2a. The scFv titers were those of the strains encoding proteins with various MFα mutations following culture in BMMY (methanol). scFv titers (bars) and biomass (final OD_660_) (circles) are indicated. Values are plotted as the mean and standard deviation from three biological replicates. Asterisks indicate significance (*p* < 0.05 by two-tailed non-paired Student’s *t*-test) for the comparison between strains encoding proteins with the wild-type and mutant MFα signal peptides.


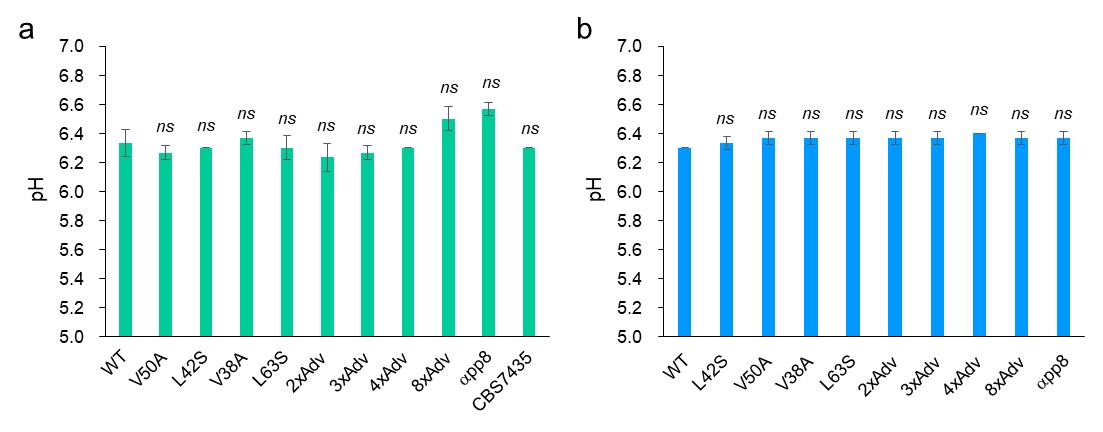


**Fig. S3 |** **pH of the culture supernatants**

pH values of culture supernatants were measured for *Pichia pastoris* strains secreting GFP (a) and BGL1p (b) that were expressed with wild-type (WT) and various MFα signal peptide mutants harboring amino acid substitutions. pH of the supernatant of the host CBS7435 strain also was measured as a control. The GFP- and BGL1p-secreting strains used were the same as those employed in Figure 3a and 3b. These strains were cultured in BMMY (methanol) for 48 h. Values are plotted as the mean and standard deviation from three biological replicates. Asterisks indicate significance (*p* < 0.05 by two-tailed non-paired Student’s *t*-test) for the comparison between strains encoding proteins with the wild-type and mutant MFα signal peptides.

**
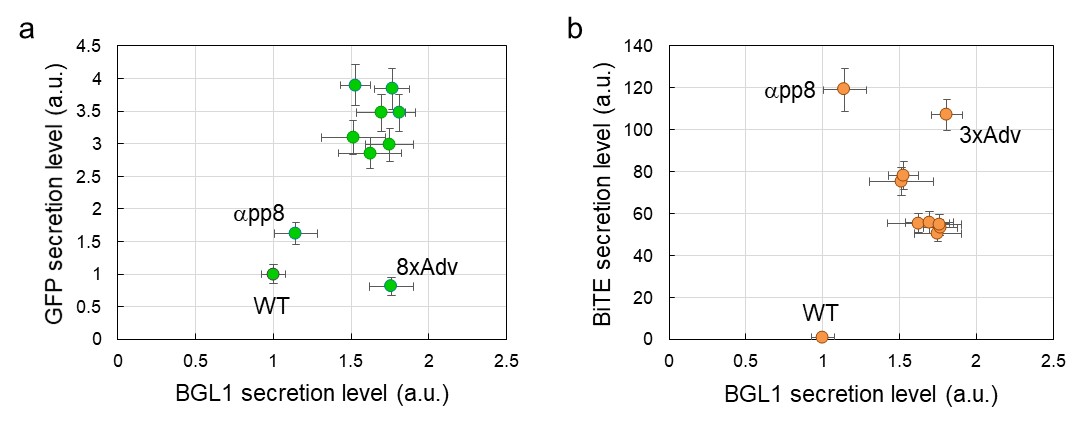
**

**Fig. S4 | Reporter gene-swap experiments.**

Relative titers (compared to the wild-type MFα signal peptide strains) for β-glucosidase (BGL1p) vs. mUkG1 (GFP) strains (a) and BGL1p vs. blinatumomab (BiTE) (b) are indicated. All strains with MFα signal peptides containing amino acid substitutions were as used in Fig. 2a, except that the αppS4 mutant was not tested. Values are plotted as mean ± standard deviation of three biological replicates.


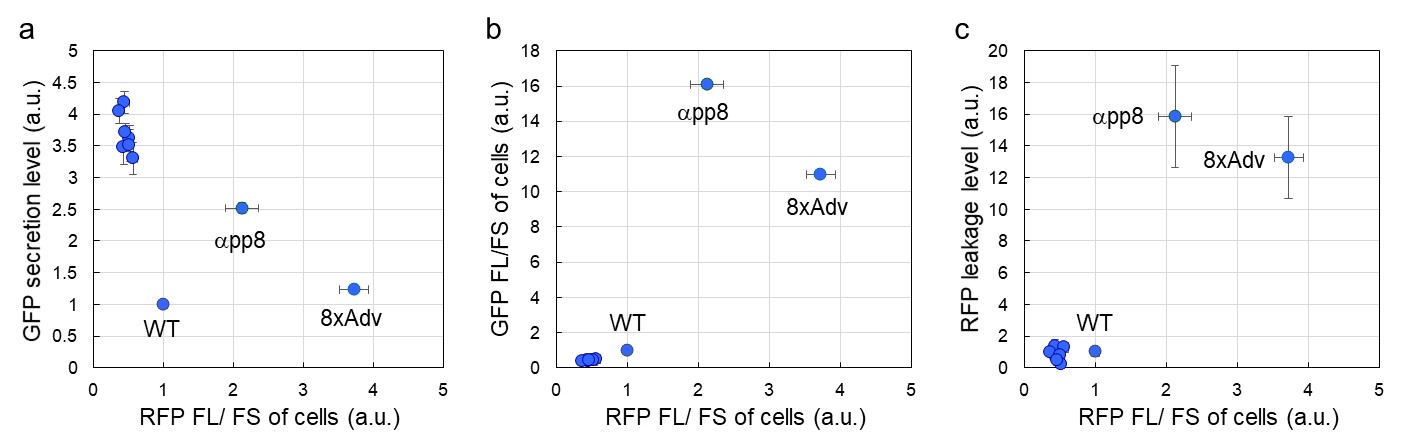


**Fig. S5 | UPR biosensor analysis of mUkG1 (GFP)-secreting strains with single and combined amino acid substitutions in the MFα signal peptide.** Cells were cultured in BMMY (methanol) medium to induce the GFP expression under the control of *AOX1* promoter. A *KAR2* promoter-*E2Crimson* (RFP)-*AOX1* terminator construct was introduced into the GFP-secreting strains. All of the MFα signal peptide amino acid substitutions were as used in Fig. 2a, except that the αppS4 mutant was not tested. The GFP and RFP fluorescence levels of the culture supernatant and the cells were measured using a microplate reader and a flow cytometer, respectively. GFP secretion (GFP fluorescence of culture supernatant) (a), remaining GFP in cells (intracellular GFP fluorescence) (b), and RFP leakage (RFP fluorescence of culture supernatant) (c) were plotted against the UPR activation levels (intracellular RFP fluorescence).


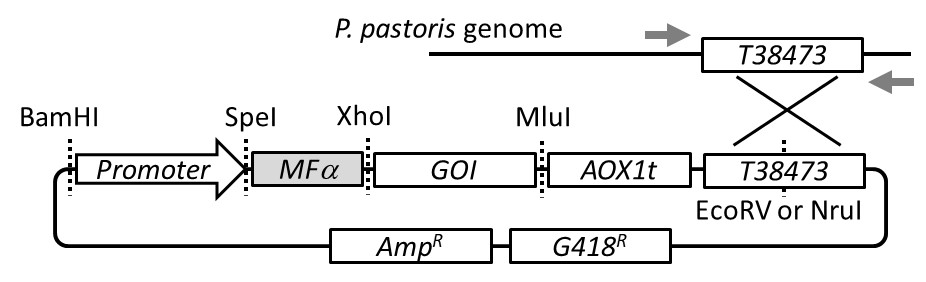


**Fig. S6 | Schematic of genome integration for construction of *Pichia pastoris* strains**

Sequences encoding wild-type and mutated MFα signals were inserted in plasmids as modules downstream of a promoter (*GAPDH* or *AOX1* promoter) and upstream of a gene of interest (GOI). In various experiments, the GOI encoded anti-lysozyme scFv, β-glucosidase (BGL1p), mUkG1p (GFP) or blinatumomab bispecific antibody (BiTE). Following linearization of the plasmid with EcoRV (or NruI) and transformation into CBS7435 (*P. pastoris* wild-type strain), homologous integration occurred in the genomic copy of the *CCA38473* (*T38473*) terminator region. A G418 resistance-encoding marker was used for selection. Gray arrows indicate positions of the primers used in colony PCR to confirm correct integration events.
